# Learning Health Systems to Bridge the Evidence-Policy-Practice Gap in Primary Health Care: Lessons From the African Health Initiative

**DOI:** 10.9745/GHSP-D-22-00390

**Published:** 2022-09-15

**Authors:** Colin Baynes, Lola Adedokun, John Koku Awoonor-Williams, Lisa R. Hirschhorn

**Affiliations:** a Department of Global Health, University of Washington, Seattle, WA, USA.; b Formerly of the Doris Duke Charitable Foundation, New York, NY, USA.; c Formerly of the Department of Policy, Planning, Monitoring and Evaluation, Ghana Health Service Accra, Ghana.; d Feinberg School of Medicine, Northwestern University, Chicago, IL, USA.; e Ryan Family Center for Global Primary Care, Havey Institute for Global Health, Northwestern University, Chicago, IL, USA.

## Abstract

The compilation of lessons in this supplement on the Doris Duke Charitable Foundation’s African Health Initiative’s work in the application of implementation research in primary health care in sub-Saharan Africa reflects the evolution of the discipline that is now increasingly recognized as integral to health systems strengthening.

## INTRODUCTION

Universal health coverage (UHC) can be achieved by strengthening the implementation of public health and clinical policies and programs in health systems.^1^ For decades, research has established a substantial evidence base on the primary health care interventions that policies should emphasize. Conversely, the need for replicable knowledge on how to transform evidence-based policies into effective large-scale action has received less attention.^2^ As a corollary, efforts to advance the use of evidence-based interventions (EBIs) in global health are replete with experiences of lengthy delays between the recognition of EBIs and their equitable and high-quality delivery throughout health systems and their integration into policies, which is needed to ensure sustainability.^3^^,^^4^ Demonstrating ways to bridge the gap between the promulgation of sound health policy and changes in real-world practice, including how systems support these changes, is essential to achieving UHC.^5^ The articles in this *Global Health: Science and Practice* supplement showcase the experience of action research projects led by policy-implementation-academic partnerships that aimed to address this gap in 3 sub-Saharan African countries: Ethiopia, Ghana, and Mozambique.

Implementation leaders can maximize the impact of EBI if they identify and implement delivery strategies that are contextually appropriate, driven by data, and feasible to use and spread within health systems. Moreover, they should include measures to ensure equity and quality of care.^6^ As the articles in this *GHSP* supplement discuss, when these strategies are combined with work to generate actionable evidence and facilitate knowledge translation, then bridging the “know-do” gap becomes increasingly attainable.^7^ For example, in Mozambique, the combination of an audit and feedback intervention led by district health officials with research capacity strengthening of health workers led by local research groups led to improvements in maternal and newborn health EBI implementation in public health facilities.^8^ Embedded implementation research (EIR) is a promising tool in this endeavor. EIR integrates scientific inquiry within practice through decision maker–led research partnerships whereby knowledge “users” (i.e., policy makers, managers, implementers) are also knowledge “producers.”^9^ As such, decision makers take a prominent role throughout the research process—identifying needs for research and specific implementation problems, selecting methodology, interpreting findings, and stimulating the use of evidence in programmatic decisions.^10^

## AHI’S HISTORY OF LEVERAGING EIR TO STRENGTHEN POLICY AND PRACTICE

Since 2009, the African Health Initiative (AHI) of the Doris Duke Charitable Foundation has funded partnerships between (1) ministries of health that led the integration of research in primary health care policy implementation, (2) embedded scientists from local research institutions that conducted EIR from within primary health care systems, and (3) local-level implementation teams that were involved in the design of delivery strategies and use of data to improve how they work. The partnership structure aimed to enable projects to leverage EIR as a tool to strengthen primary health care delivery and demonstrate ways to maximize the impact of EIR by incorporating research capacity strengthening and knowledge translation support in policy implementation processes. The AHI supported partnerships in 6 countries in 2 phases of grants: Ghana, Mozambique, Rwanda, Tanzania, and Zambia in phase 1 (2009–2015), and Ghana, Ethiopia, and Mozambique in phase 2 (2016–2022), each with grants of US$8–US$13 million. In addition, each AHI partnership included a U.S.-based university with a history of supporting health development and building research capacity in sub-Saharan Africa.

In 2013, the partnership teams co-authored a journal supplement that described their individual intervention designs and cross-cutting components, which included improving data and service delivery quality and strengthening health information systems, and a common evaluation framework that was based on the World Health Organization’s 6 building blocks for health systems strengthening.^11^^–^^15^ By 2015, key lessons emerged that were useful for informing the replication and scale of delivery strategies formulated and evaluated by the AHI phase 1 partnerships. In Ghana, the AHI project informed the Ministry of Health’s strategy on how to accelerate the pace of scaling up national primary health care policy.^16^ The Mozambican partnership strengthened district-level management capacities in the context of decentralization.^17^ In Rwanda, the project developed guidelines on quality improvement with a focus on newborns.^18^ The Tanzanian partnership designed and tested the impact of introducing a national community health worker program.^19^

These achievements were disseminated in a second AHI supplement, in which authors from the 5 teams harvested new knowledge and synthesized findings into lessons that were generalizable across partnerships. Cross-cutting effective strategies used in 2 or more countries included mentoring to improve clinical service quality and systems, improving data quality, using data for quality improvement, measuring health systems strengthening, targeting neonatal mortality, and building research capacity.^20^^–^^26^ The AHI phase 2, which focused on adapting and evaluating models to replicate effective UHC strategies from phase 1, began in 2017 when the Doris Duke Charitable Foundation granted additional funding to the teams from Ghana and Mozambique and granted new funding to a partnership in Ethiopia. The grants were also designed to strengthen the absorptive capacities of health systems to use evidence and learn about and sustain conditions that are conducive to improving primary health care performance and scaling up.

## HELPING PRIMARY HEALTH CARE SYSTEMS BRIDGE THE KNOW-DO GAP

This third supplement highlights experiences using EIR to strengthen primary health care systems. For example, authors from Ghana demonstrate how embedding research at different stages of the policy making process guided the initial design of the national Community-based Health Planning and Services program and, subsequently, how EIR-informed strategy that district implementation teams have used to accelerate the program’s scale-up.^27^ Altogether, these lessons make clear that helping health systems to acquire and foster the spread of skills to practice EIR can help fill the know-do gap.

The lessons in this supplement make clear that helping health systems to acquire and foster the spread of skills to practice EIR can help fill the know-do gap.

The articles describe the key learnings that emerged by the penultimate year of the AHI phase 2 in Ethiopia, Ghana, and Mozambique. As in the previously published supplements, the articles showcase cross-project learning that arose from collaborative working groups, which comprised AHI-partnership representatives from Ethiopia, Ghana, and Mozambique. The supplement also includes collaborative working group articles on 3 themes across the 3 countries: supportive supervision and mentoring, data use for decision making, and EIR.^28^^–^^30^ A distinguishing feature of phase 2 was the role of the Alliance for Health Policy and Systems Research (AHPSR) as an AHI partner and source of technical support to partnerships on the use of EIR. Accordingly, the supplement includes original articles on the AHPSR work in the 3 countries. From the AHPSR, Tangcharoensathien et al. also contribute a comparative piece that reflects upon the status of health policy and systems research capacity in Ethiopia and Ghana.^31^

The Ethiopia Data Use Partnership shares valuable learnings from their efforts to increase the quality and use of routine health data. Belay et al. present findings from preproject and midline data use assessments, which point to early-stage successes and challenges in strengthening routine health information systems at the point of care and the district level.^32^ Tilahun et al.’s qualitative exploration of strategies and barriers to improving the quality and use of routine data in the same settings contextualize those findings. Together, the articles yield a rich picture of the factors that underpinned early program achievements and how evidence informs strategy to improve data-use practices during the later stages of the program.^33^ Worku et al. explored whether there were preproject associations between the strength of routine health information systems and maternal health care seeking.^34^ The findings suggest patterns and frame hypotheses that Ethiopia Data Use Partnership researchers will explore at later stages of their program.

From Ghana, Awoonor-Williams et al. narrate twin histories of research utilization to inform UHC policy, comparing Ghana’s experience scaling up the national Community-based Health Planning and Services program and the National Health Insurance Scheme.^27^ Bawah et al. explore the barriers and facilitators to evidence use in policy decision making in Ghana.^35^ Both articles provide insight on how to use the EIR approach to improve the country’s UHC policy coverage and effectiveness.

From Mozambique, learnings emerged on improving management, its association with resiliency, and ongoing challenges of research use to drive needed policy. Pope et al. explore whether facility-level management capacities are associated with facilities’ readiness to provide family planning services.^36^ The findings underscore the relevance of targeting leadership structures at the facility and district level with management capacity strengthening. Inguane et al. report on lessons from embedding qualitative research in the early-stage implementation of a district-level audit and feedback strategy.^8^ Fernandes et al. performed an analysis of routine health data from 4 districts in Central Mozambique, reflecting the periods before and after Cyclone Idai in 2019.^37^ The analysis demonstrates a rapid rebound of service utilization levels in the cyclone’s aftermath, which suggests that the AHI’s investment in strengthening district management systems had a positive effect on health systems resiliency after a devastating shock. From the AHPSR, Cambe et al. illustrate the challenging context of supporting research utilization in policy contexts.^38^

The supplement concludes with perspective pieces issued by thought leaders in EIR and pri-mary health care strengthening. In their commentary, Ghaffar et al. of the AHPSR situate the findings reported in the collection of articles in the historical context in which implementation research and embedded science converged and evolved together over time.^39^ Binagwaho et al. from the University of Global Health Equity discuss the role of implementation research in establishing resilient health systems, drawing upon the authors’ experiences in Rwanda and examples from other countries.^40^

## CONCLUSION

The publication of this supplement marks a milestone in the history of the AHI and the application of implementation research in global public health. The learnings compiled in the 3 AHI collections reflect the evolution of a discipline that was relatively new to the primary health care landscape in sub-Saharan Africa when the AHI began but is now increasingly recognized as integral to health systems development. The results described in this supplement will be valuable to policy makers, researchers, and implementation teams that desire to maximize the impact of EBI through EIR and establish a culture of learning and improving in health systems. Disseminating this knowledge and supporting efforts to translate it into practice are urgently needed to hasten countries’ achievement of universal access to quality and people-centered primary health care.
